# Advancing Understanding of Chemical Exposures and Maternal-child Health Through the U.S. Environmental Influences on Child Health Outcomes (ECHO) Program: A Scoping Review

**DOI:** 10.1007/s40572-024-00456-5

**Published:** 2024-07-10

**Authors:** Emily S. Barrett, Jennifer L. Ames, Stephanie M. Eick, Alicia K. Peterson, Zorimar Rivera-Núñez, Anne P. Starling, Jessie P. Buckley, for the ECHO Cohort Consortium, Barbara O‟Brien, Lisa Peterson, Patrick Parsons, Kannan Kurunthacalam, Manish Arora, Timothy R. Fennell, Susan J. Sumner, Xiuxia Du, Susan L. Teitelbaum, Robert O. Wright, Heather M. Stapleton, P. Lee Ferguson, Akram Alshawabkeh, Judy Aschner, Clancy Blair, Leonardo Trasande, Carlos Camargo, Dana Dabelea, Daphne Koinis Mitchell, Cristiane Duarte, Anne Dunlop, Amy Elliott, Assiamira Ferrara, James Gern, Carrie Breton, Irva Hertz-Picciotto, Alison Hipwell, Margaret Karagas, Catherine Karr, Barry Lester, Leslie Leve, Debra MacKenzie, Scott Weiss, Cynthia McEvoy, Kristen Lyall, Thomas O‟Connor, Emily Oken, Mike O‟Shea, Jean Kerver, Julie Herbstman, Susan Schantz, Joseph Stanford, Leonardo Trasande, Rosalind Wright, Sheela Sathyanarayana, Anne Marie Singh, Annemarie Stroustrup, Tina Hartert, Jennifer Straughen, Qi Zhao, Katherine Rivera-Spoljaric, Emily S Barrett, Monique Marie Hedderson, Kelly J Hunt, Sunni L Mumford, Hong-Ngoc Nguyen, Hudson Santos, Rebecca Schmidt, Jonathan Slaughter

**Affiliations:** 1 Department of Biostatistics and Epidemiology, Rutgers School of Public Health; Environmental and Occupational Health Sciences Institute, Piscataway, NJ USA; 2 Division of Research, Kaiser Permanente Northern California, Oakland, CA USA; 3 Gangarosa Department of Environmental Health and Department of Epidemiology, Rollins School of Public Health, Emory University, Atlanta, GA USA; 4 Department of Epidemiology, Gillings School of Global Public Health, University of North Carolina at Chapel Hill, Chapel Hill, NC USA

**Keywords:** ECHO, Pregnancy, Chemical exposures, Children’s health

## Abstract

**Purpose of Review:**

Environmental chemical exposures may disrupt child development, with long-lasting health impacts. To date, U.S. studies of early environmental exposures have been limited in size and diversity, hindering power and generalizability. With harmonized data from over 60,000 participants representing 69 pregnancy cohorts, the National Institutes of Health’s Environmental influences on Child Health Outcomes (ECHO) Program is the largest study of U.S. children’s health. Here, we: (1) review ECHO-wide studies of chemical exposures and maternal-child health; and (2) outline opportunities for future research using ECHO data.

**Recent Findings:**

As of early 2024, in addition to over 200 single-cohort (or award) papers on chemical exposures supported by ECHO, ten collaborative multi-cohort papers have been made possible by ECHO data harmonization and new data collection. Multi-cohort papers have examined prenatal exposure to per- and polyfluoroalkyl substances (PFAS), phthalates, phenols and parabens, organophosphate esters (OPEs), metals, melamine and aromatic amines, and emerging contaminants. They have primarily focused on describing patterns of maternal exposure or examining associations with maternal and infant outcomes; fewer studies have examined later child outcomes (e.g., autism) although follow up of enrolled ECHO children continues. The NICHD’s Data and Specimen Hub (DASH) database houses extensive ECHO data including over 470,000 chemical assay results and complementary data on priority outcome areas (pre, peri-, and postnatal, airway, obesity, neurodevelopment, and positive health), making it a rich resource for future analyses.

**Summary:**

ECHO’s extensive data repository, including biomarkers of chemical exposures, can be used to advance our understanding of environmental influences on children’s health. Although few published studies have capitalized on these unique harmonized data to date, many analyses are underway with data now widely available.

## Introduction

### Background

Increasingly, the scientific community and laypeople alike recognize the powerful role that early life exposures play in shaping health and disease. Early life chemical exposures are of particular concern given mounting evidence that they may contribute to pregnancy complications, asthma and airway disease, altered growth and obesity, impacts on neurodevelopment, metabolic disease, and more [1, 2]. Exposures during gestation and early childhood may be particularly profound given the vulnerability of developing tissues and organ systems, the limited capacity to metabolize or detoxify xenobiotics, and the often higher levels of exposure compared to adults [3]. With over 8,600 chemicals currently manufactured or imported in high volume (>25,000 lbs) annually in the U.S.[4], very few of which have been comprehensively evaluated for their potential human health risks, the implications for children’s health are potentially grave [5].

The Centers for Disease Control and Prevention (CDC)’s National Health and Nutrition Examination Survey (NHANES), the leading source of U.S. biomonitoring data, makes it clear that pregnant people – and by extension their fetuses - are exposed to numerous chemicals [6]. For example, in one study, at least 43 unique chemicals were detectable in virtually every pregnant NHANES participant, with dozens more chemicals showing widespread, though not ubiquitous, exposure [7]. Highly detected chemicals included phthalates, phenols, per- and polyfluoroalkyl substances (PFAS), pesticides, and flame retardants, many with suspected endocrine disrupting, neurotoxic, and/or obesogenic impacts based on prior human and experimental literature [8].

Despite ample evidence that American pregnant people and children are exposed to a multitude of environmental contaminants, until recently, epidemiological evidence of their impacts on children’s health tended to come primarily from relatively small cohort studies focused on a single U.S. geographic area. This stands in contrast to some other countries that have successfully established large national cohorts (e.g., the Japan Environment and Children’s Study [9]) or leveraged national electronic databases and biobanks (e.g., the Danish Birth Cohort [10] or the Norwegian Mother, Father, and Child Cohort Study [11]) to study children’s environmental health. In the early 2000s, the U.S. National Children’s Study was initiated to remedy this gap [12], but its failure to launch on a national scale led researchers to rethink the study design and consider drawing upon established strengths, expertise, and infrastructure [13].

With that in mind, in 2016, the National Institutes of Health (NIH) embarked upon the Environmental influences on Child Health Outcomes (ECHO) Program, the largest ever U.S. study of how environmental factors may influence children’s health and development [14]. ECHO’s goals were ambitious: to harmonize extant data and collect new data on >50,000 U.S. children from 69 existing pregnancy and child cohorts across the country [15]. In doing so, the program would create a single ECHO cohort with the power and diversity to address some of the most pressing issues in children’s health. In 2020, as efforts to harmonize relevant data were actively underway, we reported on opportunities for ECHO to advance the field, describing existing data and plans for new data collection [16]. Four years later, with data and biospecimens now available for use by the scientific community at large, we take stock of ECHO’s progress to date regarding chemical exposures and maternal-child health. First, we review the published ECHO literature, highlighting papers including data on biomarkers of chemical exposures from participants across multiple ECHO awards. Second, we discuss future directions for the ECHO Program as well as opportunities to use existing ECHO data and biospecimens for future analyses.

### ECHO Overview and Structure (2016-2023)

ECHO was launched in 2016 as an NIH extramural program. In addition to its interventional arm (the IDeA States Pediatric Clinical Trials Network [ISPCTN]), ECHO developed an observational “cohort of cohorts” consisting of 69 pre-existing longitudinal cohort studies focused on children’s health [14]. The goals were to: (1) facilitate impactful research to inform practice and policy around children’s health and development; and (2) create a data and biospecimen repository for pediatric research in the U.S. Five main outcome areas were prioritized: (1) pre-, peri-, and postnatal outcomes; (2) upper and lower airways; (3) obesity; (4) neurodevelopment; and (5) positive health. By leveraging existing cohorts, the program could capitalize on established infrastructure and expertise as well as ongoing relationships and trust between the local cohorts and their participants. In total, over 60,000 children from around the U.S. contributed data in the first seven-year cycle of ECHO [15].

A major effort during the first cycle of ECHO was to harmonize existing data from the cohorts to facilitate ECHO-wide pooled statistical analyses. This included data on the priority outcome areas as well as relevant exposure and covariate data. Of particular relevance to the current review, protocols were developed to transfer and harmonize existing chemical biomarker data that were generated by numerous labs over several decades, contending with challenges around inter-lab differences, temporal trends, and more. In addition to harmonizing extant data, all cohorts implemented the ECHO-Wide Cohort Protocol (EWCP) to collect common data elements and biospecimens in a standardized fashion moving forward. The EWCP included questionnaires on sources of chemical exposures, extensive residential address histories to facilitate linkages with geospatial data, and common procedures for collecting biospecimens including maternal and child blood and urine. New chemical analysis of ECHO biospecimens was conducted in conjunction with the NIEHS Child Health Exposure Analysis Resource (CHEAR) [17] and Human Health Exposure Analysis Resource (HHEAR) [18] programs with considerable investigator input on priority exposures to be studied. The HHEAR labs developed assays in response to these identified priorities with an emphasis on multi-chemical panels that provide extensive data while conserving biospecimen volume (e.g., PFAS, organophosphate esters (OPEs), multiclass chemicals). In parallel, the ECHO Data Analysis Center (DAC) developed protocols and guidance on the harmonization of biomarker data generated by different labs or based on slightly different protocols. In this review, we focus on published ECHO studies that include data on biomarkers of chemical exposure from more than one ECHO award, to emphasize the opportunities that ECHO has created for efficient, collaborative, high-impact research.

## Methods

### Identification of relevant ECHO-wide papers

Relevant papers were identified through the ECHO Program Publications website, which is publicly available and updated regularly (https://echochildren.org/echo-program-publications/) [19]. It includes all publications that cite funding from one or more ECHO awards (individual grants). Using the record of all publications through December 31, 2023 as a starting point, all seven authors of this review used a structured approach, evaluating titles and abstracts to first identify papers that: (1) focused on chemical exposures; (2) were developed as part of the ECHO Program; and (3) included data from ECHO participants. We evaluated papers considering early-life chemical exposures including PFAS, phthalates/phthalate alternatives, phenols, OPEs, metals/metalloids, pesticides, fungicides, herbicides, disinfection byproducts, polycyclic aromatic hydrocarbons (PAHs), perchlorate, polybrominated diphenyl ethers (PBDEs), polychlorinated biphenyls (PCBs), aromatic amines, melamine and melamine derivatives, tobacco metabolites, and other environmental chemicals. Given our focus on biomarkers of chemical exposures, rather than environmental contaminants more generally, we did not include papers that focused on air pollutants or other geospatially-derived chemical exposure data [e.g., 20, 21, 22]. For papers meeting the criteria above, based on the abstracts and/or full texts, we then abstracted data including the exposure, outcome, life stage, and type of paper (e.g., review, methods, original data analysis), determining which papers included ECHO participants from multiple awards (individual funded grants) and would therefore be discussed in greater detail (see ***Multi-award papers***). Of note, there were several individual ECHO grant awards that included multiple ECHO cohorts. Papers that were based solely on participants from the cohorts within those awards were not included in the current review as our priority was to highlight the national-level analyses conducted using the central ECHO infrastructure. Prior to publication, we revisited the ECHO publications website to identify any new papers meeting all criteria above that were published through March 1, 2024.

## Results

### Overview of Search Results

**Figure 1.**
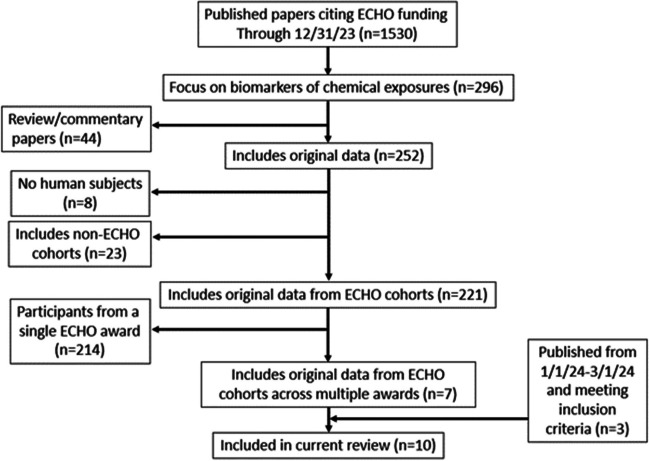
Overview of process to identify relevant papers

**Figure 2.**
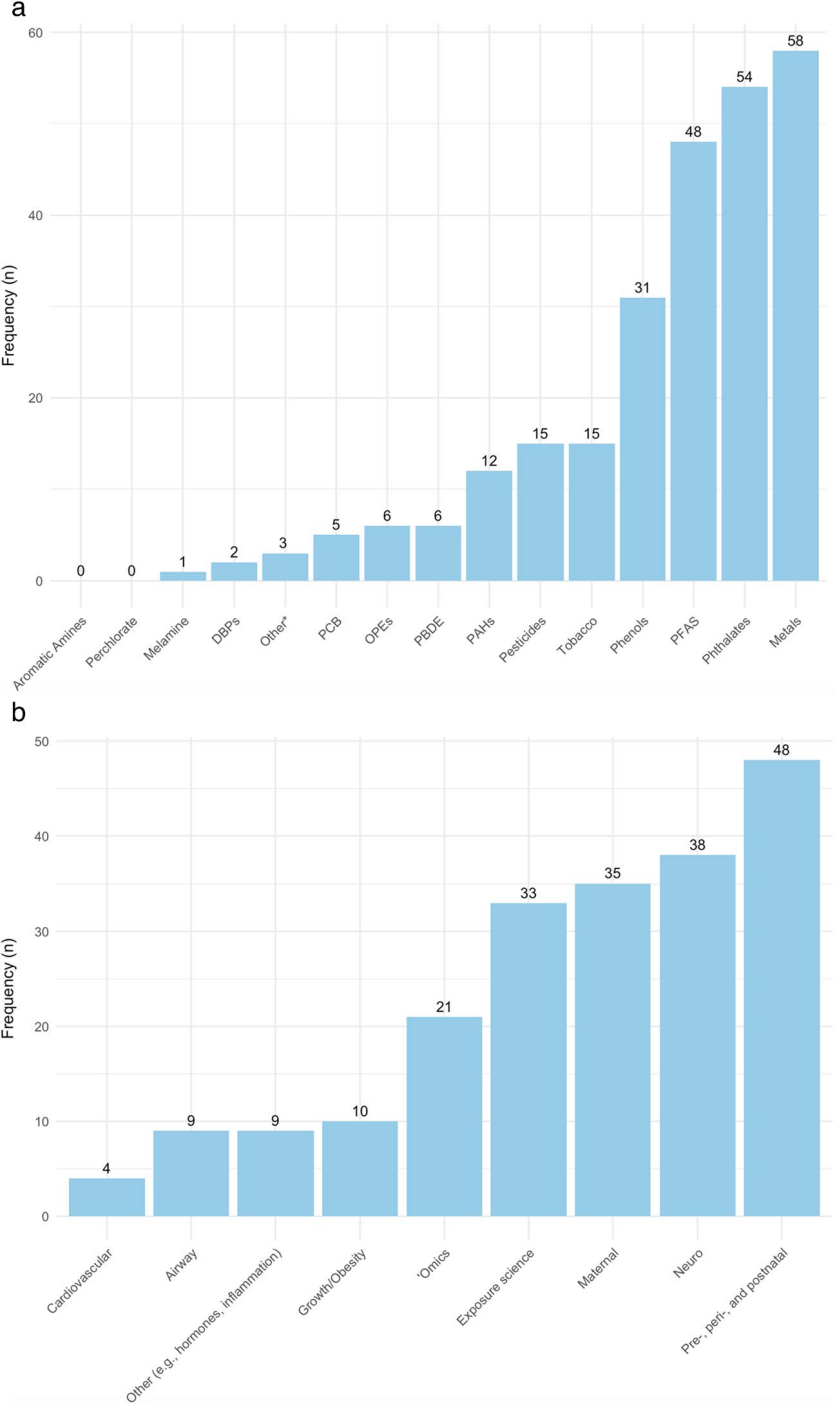
Single award ECHO papers examining chemical exposures through 12/31/23 (n=214) by (2a) chemical group; and (2b) outcome of interest

In total, through December 31, 2023, 1,530 papers were published acknowledging ECHO funding (Figure 1). Of those, 296 were considered focused on biomarkers of chemical exposures. We excluded 44 review papers from further consideration as well as 8 papers that did not include human participants and 23 papers that were based on non-ECHO cohorts. Of the remaining 221 papers, 214 were based on participants from a single ECHO award (which in some cases was comprised of more than one cohort). The single-award papers focused on a variety of exposures (Figure 2a) and outcomes (Figure 2b), reflecting the wide breadth of ECHO data and investigator expertise.

In total, based on the initial search, we identified seven multi-award papers meeting our criteria. A follow-up search for papers published between December 31, 2023 and March 1, 2024 yielded three additional multi-award papers, for a total of ten papers eligible for inclusion.

### Multi-Award Papers

#### PFAS

Three ECHO-wide analyses assessed the association of prenatal PFAS exposure with childhood growth and development outcomes, all incorporating statistically advanced methods for estimating the effects of exposure to chemical mixtures. PFAS data used in these analyses came from multiple labs including HHEAR, the Centers for Disease Control (CDC), and the California Department of Toxic Substances Control; of note, all labs contributing data were part of the CDC’s inter-laboratory quality assurance program. Padula et al. (2023) examined the role of prenatal exposure to PFAS on birth outcomes and the potential modification of associations by maternal perceived stress during pregnancy [23]. The analysis included 3,339 mother-child pairs from 11 ECHO cohorts. PFAS were measured in maternal plasma or serum collected during pregnancy and included perfluorooctanesulfonic acid (PFOS), perfluorooctanoic acid (PFOA), perfluorononanoic acid (PFNA), perfluorohexanesulfonic acid (PFHxS), and perfluorodecanoic acid (PFDA) with >60% of samples above the limit of detection (LOD). Outcomes included gestational age at birth (weeks), preterm birth (<37 weeks vs. ≥37 weeks), birth weight for gestational age z-scores, and small for gestational age (SGA, <10th percentile) and large for gestational age (LGA, >90th percentile). Maternal stress as an effect modifier was assessed by the Cohen’s Perceived Stress Scale [23]. Results indicated inverse relationships between prenatal PFAS exposure and birth weight. For each one-unit of exposure (log-transformed) to PFOA, PFOS, PFNA, and PFDA, lower birth weight-for-gestational-age z-scores were observed: β: -0.15 (95% CI: -0.27, -0.03), β: -0.14 (95% CI: -0.28, -0.002), β: -0.22 (95% CI: -0.23, -0.10), and β: -0.25 (95% CI: -0.37, -0.14), respectively. Correspondingly, there was a decreased odds ratio (OR) for large-for-gestational-age in relation to PFNA (OR: 0.56; 95% CI: 0.38, 0.83), and PFDA (OR: 0.52; 95% CI: 0.35, 0.77). In the summed effect models (Bayesian Weighted Sums), PFDA was the strongest contributor to the summed effect. The study found no evidence that maternal perceived stress modified PFAS exposure and birth outcomes within the ECHO cohort.

A second study assessed prenatal PFAS exposure with childhood body mass index (BMI) trajectories and the risk of overweight/obesity in 1,391 mother-child pairs from 8 ECHO cohorts [24]. The study evaluated 7 PFAS (PFOA, PFOS, PFNA, PFHxS, PFDA, perfluroundecanoic acid (PFUnDA), and N-methylperfluorooctane sulfonamidoacetic acid (NMFOSAA)), all with at least 50% of samples above the LOD and measured in maternal serum or plasma collected during pregnancy. Heights and weights measured between 2-5 years of childhood were used to calculate age- and sex-specific BMI z-scores, with overweight/obesity defined as >85th percentile. A doubling of PFHxS was associated with higher child BMI z-score (β: 0.07; 95% CI: 0.01, 0.12), while each doubling of PFUnDA and PFOS was associated with an increased risk of overweight/obesity (PFUnDA Risk Ratio (RR): 1.10; 95% CI 1.04, 1.16, PFOS RR: 1.12; 95% CI 1.01, 1.24). Results for NMFOSAA were similarly suggestive for risk of overweight/obesity (RR: 1.06; 95% CI 1.00, 1.12). There was no evidence for modification by child sex. In co-pollutant models using semi-Bayes and Bayesian weighted sums for PFAS mixtures, PFHxS and PFNA were most strongly associated with BMI z-scores.

The third published ECHO-wide analysis utilizing prenatal PFAS exposure investigated associations with child autism-related outcomes as measured using the Social Responsiveness Scale (SRS) [25] and clinical diagnosis of autism [26]. They also evaluated potential effect modification by child sex. The study was comprised of 1,429 mother-child pairs from ten cohorts. Eight PFAS were included (>50% of samples >LOD): PFOA, PFOS, PFNA, PFHxS, PFDA, PFUnDA, NMFOSAA, and 2-(N-Ethyl-perfluorooctane sulfonamido) acetic acid (EtFOSAA) from maternal serum or plasma collected during pregnancy. PFAS mixtures were evaluated using Bayesian methods (semi-Bayes and Bayesian weighted sums). Overall, prenatal PFAS exposure was not associated with child SRS T-scores nor autism diagnosis, though there was a suggestive positive association between PFNA exposure and greater autistic traits as measured by the SRS T-score (β: 1.5 points per ln increase; 95% CI: 0.1, 3.0). No consistent evidence was found within the summed mixture model, nor for sex-specific results.

##### Phthalates/Phenols/Parabens

To date, three ECHO multi-award studies have examined associations between prenatal exposure to phthalates and/or environmental phenols and maternal and child health outcomes. Of those, two papers by Trasande et al. examined prenatal exposure to phthalates and phenols in relation to birth outcomes [27, 28]. In the paper on phthalate exposure, 20 phthalate metabolites were measured by the HHEAR and CDC labs in maternal urine during pregnancy in 13 ECHO cohorts, resulting in a sample size of 5,006 mother-child pairs [28]. In the primary analyses, phthalate metabolites were analyzed as molar sums based on typical use categories (high molecular weight vs. low molecular weight) and parent compounds. Birth outcomes were considered continuously (gestational age at birth, birth weight, birth length, and birth weight-for-gestational-age z-scores) and categorically (preterm birth, low birth weight, small for gestational age, and large for gestational age). Stratified analyses were conducted to detect possible effect modification by child sex, maternal education, parity, and race/ethnicity. Multiple phthalate metabolites were associated with lower gestational age at birth and birth weight, and with greater odds of preterm birth and low birth weight. Generally, the magnitude of association was greater for metabolites of DiNP, DiDP, and DnOP compared to the other molar sums examined. For example, DiNP (OR: 2.25; 95%CI: 1.67-3.00), DiDP (OR: 1.69; 95%CI: 1.25, 2.28), and DnOP (OR: 2.90; 95%CI: 1.96, 4.23) were all associated with increased odds of preterm birth. There were no associations with birthweight-for-gestational-age, suggesting that observed birth weight effects may be mediated by the duration of gestation. The authors additionally estimated the financial costs of excess preterm births in the U.S. that could be attributed to phthalate exposure (assuming a causal relationship) as $3.84 billion in 2018 alone.

The second paper by Trasande et al. investigated prenatal exposure to environmental phenols and parabens in relation to infant birth outcomes [27]. This analysis included 11 ECHO cohorts and 3,619 mother-child pairs. Exposures were measured in maternal urine during pregnancy at the HHEAR, CDC, and California Department of Public Health and the outcomes were the same as in the previous analysis of phthalate exposure. Of the 11 chemicals evaluated, most were not associated with birth weight or gestational age at birth, with a few exceptions. Benzophenone-3, used as a UV filter [29], was associated with lower birth weight (β: -29.21g per log_10_-unit increase; 95% CI: -58.03, -0.40) and lower birth weight-for-gestational-age. Methyl paraben was associated with lower birth weight-for-gestational-age z-score (β: -0.10 SD units; 95% CI: -0.18, -0.02). Notably, no associations with bisphenol A were detected; however, bisphenol A levels tended to be lower than in previous studies [30].

The third study, by Jacobson et al., investigated how multiple classes of chemicals found in consumer products (phthalates, phenols, parabens, and triclocarban) may be associated with maternal postpartum depression [31]. This analysis included 5 cohorts and 2,174 participants. The chemical exposures were measured in maternal urine collected during pregnancy by the CDC or HHEAR. The analysis was restricted to the subset of chemicals that were measured in 3 or more cohorts and detected in >50% of participant samples. Postpartum depression was defined based on harmonized scores from two widely used, validated depression screening tools: the Edinburgh Postnatal Depression Scale (EPDS) and the Center for Epidemiologic Studies Depression Scale (CES-D). Scores from both instruments were harmonized to the Patient-Reported Measurement Information System (PROMIS) depression scale for continuous analyses [32]. Additionally, two separate thresholds were used to classify participants as having postpartum depression: one with greater sensitivity (EPDS ≥ 10 / CES-D ≥ 16) and one with greater specificity (EPDS ≥ 13 / CES-D ≥ 20). The prevalence of postpartum depression in this population ranged from 8% to 16% depending on the definition used. There were no significant associations between chemical exposures and continuous PROMIS scores. Prenatal high molecular weight phthalate metabolite concentrations were associated with higher odds of postpartum depression when using the more sensitive definition (OR: 1.11; 95%CI 1.00-1.23) and the association was similar, but slightly attenuated, when using the more specific definition.

##### OPEs

Though organophosphate ester flame retardants and plasticizers (OPEs) have been in use for several decades, their production rose dramatically to replace the industry phase out of polybrominated diphenyl ethers (PBDE) flame retardants in the early 2000s [33]. In ECHO, nine OPE metabolites were measured in single spot or morning void urine samples collected between the 2^nd^ and 3^rd^ trimesters from 7,048 mothers across 16 cohorts, representing the largest sample to date with these pregnancy measurements. In contrast to some other ECHO-wide studies in which existing chemical assay data from multiple labs was harmonized, for this analysis, all OPEs were analyzed *de novo* at a single HHEAR laboratory [34]. OPE metabolites had varying levels of detection in the sample: diphenyl phosphate (DPHP), a composite of dibutyl phosphate and di-isobutyl phosphate (DBUP/DIBP), and bis(1,3-dichloro-2-propyl) phosphate (BDCPP) were detected in >87% of study samples; bis(2-chloroethyl) phosphate (BCETP), bis(butoxyethyl) phosphate (BBOEP), and BCPP were detected in 50-80%; and bis(2-methylphenyl) phosphate (BMPP), bis(2-ethylhexyl) phosphate (BEHP), and dipropyl phosphate (DPRP) were detected in <36%. Results indicated that higher levels of several prenatal OPEs were adversely associated with gestational duration and fetal growth [35]. Specifically, DBUP/DIBP (OR per doubling: 1.07; 95%CI: 1.02, 1.12) and BBOEP (OR in high group vs non-detect: 1.25; 95%CI: 1.06, 1.46) were associated with higher risk of preterm birth. Furthermore, several OPEs were associated with shorter gestational length and preterm birth among female children only. Mothers with higher levels of BCPP, BMPP, and DPRP were also more likely to have babies with higher birth weight-for-gestational age, a potential precursor to childhood obesity. Additional studies in ECHO examining OPEs in relation to neurodevelopment and childhood obesity are currently underway.

##### Metals

Howe et al. (2022) examined prenatal metal mixtures and birth weight for gestational age in a pooled analysis of three ECHO cohorts (n=1,002) [36]. Seven metals commonly measured in maternal urine collected during pregnancy were evaluated: antimony (Sb), cadmium (Cd), cobalt (Co), mercury (Hg), molybdenum (Mo), nickel (Ni) and tin (Sn) by HHEAR or the Dartmouth Trace Element Analysis Core. Investigators did not observe associations between the overall metal mixture and birth weight using Bayesian kernel machine regression (BKMR) modeling. However, they reported associations between individual metals and birth weight after adjusting for co-exposure to other metals in the mixture. For example, inverse associations with birth weight were identified for Hg, Sb, and Sn, while a positive association was identified for Ni, and a reverse j-shaped association was identified for Co. A second ECHO-wide metals study which focused on maternal arsenic exposure and birth outcomes in 15,342 dyads was published in 2023 but is not discussed further here as exposure was estimated based on data from public water systems rather than a biomarker [22].

##### Other/multiclass panels

In addition to the above chemical classes that are routinely included in NHANES biomonitoring, ECHO investigators conducted an extensive review to identify and prioritize new chemicals for biomonitoring [37]. A total of 155 chemicals were thoroughly evaluated for likelihood of exposure, potential toxicity, and existence of a biomarker, and 36 were recommended for biomonitoring in ECHO. Based on this evaluation, a biomonitoring study to measure priority contemporary and emerging chemicals in pregnancy urine samples from 171 participants in 9 ECHO cohorts was launched through the HHEAR lab [38, 39]. The study used a novel multiclass analytical chemistry approach to measure many of the recommended chemicals as well as other chemicals of concern in a single urine sample, simultaneously quantifying 89 analytes across 9 chemical classes including bactericides, benzophenones, bisphenols, fungicides and herbicides, insecticides, OPEs, parabens, phthalates/alternative plasticizers, and polycyclic aromatic hydrocarbons (PAHs) [38]. Of the 89 analytes, 73 were detected in at least one sample and 35 were detected in over half of participants. Notably, five of the widely detected analytes (benzophenone-1, thiamethoxam, mono-2-(propyl-6-carboxy-hexyl) phthalate, monocarboxy isooctyl phthalate, and monohydroxy-iso-decyl phthalate) are not currently included in NHANES biomonitoring. A follow-up study in the same pregnant ECHO participants evaluated urinary concentrations of melamine, three melamine derivatives, and 39 aromatic amines through the HHEAR lab [39]. Sixteen analytes were detected in at least one sample, with melamine, its derivative cyanuric acid, and 9 aromatic amines detected in >60% of participants. Across both studies, concentrations of multiple chemical biomarkers were higher among Hispanic/Latina and non-Hispanic Black participants compared to non-Hispanic White participants. Both studies also reported notable trends in concentrations over time; for example, there were decreasing concentrations of several phthalate metabolites but increasing concentrations of the phthalate replacement di-iso-nonyl-cyclohexane-1,2-dicarboxylic acid (DINCH) [38]. Based on widespread detection of these chemicals, ECHO launched a larger biomonitoring study (currently underway) of over 6,000 pregnancies using an expanded multiclass assay of contemporary and emerging chemicals and over 1,700 pregnancies with melamines and aromatic amines to facilitate research examining the children’s health effects of these understudied chemicals.

## Discussion

### Summary

With ECHO’s extensive data harmonization efforts combined with implementation of standardized data collection and new biomarkers measurements, the cohort is now advancing large studies of environmental chemicals on children’s health. ECHO’s first multi-award papers have examined PFAS, phthalates, phenols and parabens, OPEs, metals, melamine and aromatic amines, and emerging contaminants. They have primarily focused on describing patterns of maternal exposure or examining associations with maternal and infant outcomes; studies on later child outcomes (e.g., autism, obesity) are fewer, but are accelerating as the cohort ages and data become available.

### Use of current ECHO chemical exposures data

#### Accessing data

ECHO is a resource for the entire scientific community with data available to investigators within and outside of ECHO. A restricted (de-identified) version of the ECHO-wide Cohort data is available by request from the NICHD Data and Specimen Hub (DASH) [40]. ECHO datasets available through the 2^nd^ DASH release (through August 31, 2022) contain data on 63,215 ECHO participants. In addition to rich information on demographics, pregnancy characteristics, and child health outcomes for ECHO participants, there are 471,387 bioassay results for chemical exposures. Most of the bioassays were of samples collected during pregnancy (79%), with the vast majority in urine (84%), followed by blood (14%). Less than 1% of chemical assays were conducted in hair (n=2,043), meconium (n=4,308), saliva (n=407), umbilical cord blood (n=1,688), or umbilical cord tissue (n=2,409). As shown in Table 1, the greatest amount of information is available for pregnancy exposures to metals and metalloids, OPEs, PFAS, environmental phenols, and phthalates. The DASH dataset is updated as additional assay results are available, providing a unique resource to study chemical exposures and children’s health.

**Table 1.** Chemical bioassay data available in the NICHD Data and Specimens Hub (DASH), ECHO-wide Cohort 2^nd^ Release (through 8/31/22).

Assay Class	Number of analytes in the class with DASH data	Number of unique results	% of samples from pregnancy
Alkyl Phosphate Pesticides (Organophosphorus Insecticides) and Pyrethroids	10	7064	70
Aromatic Amines	39	4992	100
Fungicides and Herbicides	11	1584	100
Insecticides, not otherwise specified	1	144	100
Melamine and Melamine Derivatives	4	580	100
Metals and Metalloids	34	61269	79
Neonicotinoid Insecticides	11	1584	100
Organophosphorus Flame Retardants	17	39136	100
Perfluoroalkyl and Polyfluoroalkyl Substances	21	49570	76
Environmental Phenols	32	94750	72
Phthalate and Phthalate Alternatives	44	196303	80
Polycyclic Aromatic Hydrocarbons (PAHs)	11	3752	15
Tobacco metabolites	5	10659	82

Note: The DASH release also includes results for specific gravity and creatinine to account for urinary dilution (n=21,482)

### Opportunities for innovative analyses of chemical exposures data

The growing size, diversity, and comprehensiveness of ECHO resources presents unique opportunities to advance our understanding of who is most vulnerable to specific chemical exposures and how these exposures influence child health. First, ECHO spans a wide range of birth years (dating back to the 1980s) and geography (spanning 49 U.S. states, the District of Columbia, Puerto Rico, and the Navajo Nation) which can be harnessed to examine temporal and spatial trends in exposure [15]. ECHO additionally includes participants from the Navajo Nation as well as from both rural and urban settings. The potential to identify exposure vulnerabilities by geographic region has been demonstrated in ECHO-wide studies of arsenic in drinking water [22] and air pollution [21] but has not yet been investigated for other pollutants. Furthermore, the capability to link across other geospatial exposure data, including these water contaminant and air pollution databases, as well as neighborhood-level factors such as the social vulnerability index (SVI) [20, 21, 41] and child opportunity index (COI) [20, 21, 42], offers the ability to examine not only joint effects of chemical and social stressors but also paint a more complete portrait of a person’s exposome and their cumulative exposure burdens. The biomarker data that was the focus on the current analysis, furthermore, is complemented by questionnaire data that may provide insights on sources of exposure [e.g., 43]. Additional novel directions include:

- *‘Omics.* Currently, ECHO is collecting genetic and epigenetic data on mothers and children. These data can be layered with metabolomic and potentially other ‘omic data, creating a rich research resource for understanding the biological underpinnings of the developmental toxicity of environmental chemicals. Individual cohorts within ECHO have already undertaken such analyses, including studies of PFAS exposures and the prenatal and neonatal metabolome [44, 45]. It will be important to validate and expand these studies in the larger ECHO-wide cohort. ECHO’s size and genetic data will also be fertile ground for the still nascent field of gene-by-environment research, which has been historically limited by a lack of large study samples with both rich environmental and genetic data in diverse populations.
- *Mixtures.* The large sample size and rich exposure biomarkers in ECHO also afford opportunities to ask key questions about how exposures to complex mixtures both across and within classes of chemicals affect child health. To date, ECHO-wide studies have implemented a variety of statistical approaches for the analysis of chemical mixtures, depending on the specific research question and the structure of the exposure data [23, 24, 26, 36]. These have included Quantile G-Computation, BKMR, Bayesian Weighted Sums, and burden scores, though many other approaches could also be suitable within the ECHO dataset. As more investigators pursue these questions about cumulative and synergistic health effects of multiple chemical exposures, it will be important to consider the correlations among chemical biomarkers and to incorporate knowledge about shared environmental sources and biological modes of action among the mixture components.
- *Emerging contaminants.* In addition to the chemicals addressed in this review, ECHO’s exposure biomarker data will continue to expand to address emerging contaminants. For example, a multiclass assay of >100 contemporary and emerging non-persistent chemicals in mid-pregnancy urine has just been completed in over 6,000 ECHO participants across 23 cohorts. While this is only a subsample of ECHO participants, it presents a test case of how ECHO’s size and biospecimens can be leveraged to accelerate our understanding of how emerging chemical hazards impact children’s environmental health. As ECHO continues biospecimen collection in participants as children age and expands the sample to include new pregnancy recruitment sites in the second cycle (2023-2030), the multiclass chemicals subsample will be a key resource to monitor and study emerging chemicals of concern.
- *Role of postnatal exposures.* While ECHO efforts to date have focused on biomarkers of chemical exposures measured in maternal samples collected during pregnancy, it is important to highlight that chemical exposures are ongoing throughout infancy, childhood, adolescence, and adult life. Chemical exposures occurring during potentially vulnerable life stages (e.g., infancy, puberty) may impact subsequent health and development and may also compound the impacts of prenatal exposures (i.e., a “two-hit” model). Moreover, correlations between prenatal and postnatal exposures should be carefully examined, to better understand whether observed associations with child health may reflect confounding by childhood exposure rather than a true effect of exposures during the prenatal period.
- *Disparities.* ECHO has prioritized the engagement of diverse families to better approximate the U.S. population. As of March 2022, 59.9% of ECHO children were White, 16.1% were Black, 12.1% reported multiple racial identities, 3.2% were Asian, 3.1% were American Indian or Alaska Native, 0.4% were Native Hawaiian or Pacific Islander, and 4.9% identified as belonging to another racial group. Additionally, a quarter (25.8%) identified as being of Hispanic ethnicity [15]. These proportions are expected to rise in the second cycle as an even greater emphasis is placed on engaging diverse participants. The cohorts are also diverse in terms of socioeconomic and geographic factors, all design features that will allow investigators to better understand disparities in chemical exposures in U.S. mothers and children. Already, several ECHO-wide papers have highlighted racial and ethnic disparities in levels of chemical exposures [38, 39] as well as differences in their associations with adverse outcomes [27, 28].

### New directions in ECHO (2023-2030)

What started as a seven-year initiative has now been extended for at least another seven years (Cycle 2, 2023-2030) with new infrastructure and opportunities to advance the science on early life chemical exposures and children’s health [46]. Forty-nine new awards were made in 2023 to enable continued follow-up of 30,000 current ECHO children and recruit 30,000 new pregnant people across the country. In this new cycle of ECHO, the focus is squarely on science that makes use of ECHO-wide data (termed “ECHO Cohort” data in Cycle 2) rather than site-specific analyses. To that end, new data collection has been further standardized across sites, which will all implement a single protocol. In contrast to the “cohort of cohorts” in ECHO’s first cycle, the new cycle is best seen as a single cohort with numerous data collection sites across the U.S. The ECHO protocol now includes two prenatal visits, one perinatal, and two infant visits, followed by annual visits across childhood. Visits are designed to facilitate both in person and remote data collection as needed. In Cycle 2, biospecimens will be managed and analyzed by the centralized ECHO Lab Core (“ELVIS”) based at Vanderbilt University Medical Center. Additional noteworthy new study components in the next phase of ECHO include:

- *Recruitment of a pre-conception cohort.* Participants who deliver in the second cycle of ECHO will be invited to continue their participation in the postpartum period. Given that many such participants will likely go on to have another pregnancy, the data collection between the first ECHO delivery and the subsequent pregnancy is effectively pre-conception data, which will facilitate novel research on how chemical exposures may impact fecundity, early fetal development, and pregnancy loss.
- *Conceiving partners.* In contrast to the first cycle of ECHO wherein only indirect, maternal-reported data on conceiving partners was captured, in the second cycle, those partners will be invited to enroll in the study and provide self-reported data and biospecimens. Studying these “triads” including both conceiving partners and the child may yield insights into the relative contributions of maternal and paternal factors to children’s health and development and provide opportunities to measure biomarkers of chemical exposures in novel biospecimens (e.g., semen).
- *Specialized protocols.* Recognizing the tension between assessing the myriad exposures and outcomes that are relevant to children’s health and concerns about participant burden, the vast majority of ECHO data collection moving forward comes from implementation of a single streamlined, standardized ECHO cohort protocol implemented by all study sites. However, in addition to that standardized protocol, each site will implement complementary, specialized protocols aligned with their team’s particular interests and expertise. The specialized protocols include exposure areas (physical and chemical, lifestyle, psychosocial) as well as outcome areas (pre-, peri-, and postnatal, upper and lower airways, obesity, and neurodevelopment) and each will consist of no more than 30 minutes of additional focused data collection at each study visit in a subset of the 60,000 participants. While the specialized protocols are still under development, we can anticipate that they may include biospecimen collection, environmental samples, questionnaires, and direct assessments. For instance, a physical and chemical exposures specialized protocol might include additional questionnaire items on sources of exposure (e.g., personal care products) as well as additional biospecimens (e.g., breast milk) or collection of water samples that are not part of the main protocol.

### Strengths and Challenges

#### Strengths

With over 60,000 participants, ECHO’s size provides unprecedented power to examine the impacts of chemical exposures on maternal and child health in the U.S. This includes providing definitive confirmation of previously reported trends as well as investigating impacts of new and emerging chemicals. ECHO’s geographic and sociodemographic variation can illuminate sources of exposures and identify particularly vulnerable subpopulations. Given that prenatal enrollment into the original cohorts spanned multiple decades and the intensive, sustained follow-up of participants, ECHO is well-positioned to examine how ever-evolving societal dynamics and chemical exposures policy as well as the introduction of new replacement chemicals impact perinatal and children’s health outcomes. ECHO’s infrastructure has been built to support research on a wide range of chemical exposures including contemporary and new chemicals, mixtures, and multiclass panels using chemical analytical protocols. The more recent harmonization of analytical protocols and the move towards standardized sample collection will further strengthen ECHO analyses of chemical exposures moving forward. Finally, the existent robust infrastructure is responsive to new and rapid-occurring exposures (e.g., the COVID pandemic) ensuring the integrity of data collection as well as the potential to study the impacts of these natural experiments on chemical exposures and child health outcomes [43, 47]. Moving forward, ECHO presents opportunities to develop and test dissemination and implementation strategies to reduce chemical exposure. For example, inclusion of the preconception period on ECHO cycle 2 can provide an opportunity to implement reduction strategies for chemicals such as phthalates and measure the impact of those strategies.

#### Challenges

At the same time, compared to traditional single cohort approaches, the complexity of ECHO presents some notable challenges with respect to chemical exposures data. ECHO’s rich database of chemical bioassay results combines information collected by cohort sites prior to the onset of ECHO as well as assays funded by ECHO through HHEAR [15, 48]. Combining data generated over several decades by many laboratories based on samples collected by multiple sites following different protocols results in harmonization challenges. Among these issues are differences in biospecimen collection, processing, and storage (e.g., matrix type, freeze-thaw cycles, storage temperature, length of storage, fasting status, timing of collection) and inter-laboratory differences (e.g., analytical chemistry methods, limits of detection, assay reliability, quality control procedures). The ECHO Data Analysis Center (DAC) established a detailed protocol for obtaining bioassay data from cohorts to capture these important details and facilitate harmonization of chemical biomarker information. While inter-cohort and inter-laboratory differences exist, the systematic capture of differences in biospecimen collection, storage, and processing across cohorts (e.g., in fasting status, sample additives, storage temperature, or freeze-thaw cycles) allows investigators to interrogate the extent of these differences in the chemical data and conduct sensitivity analyses as needed to determine their impact on analysis results. The DAC has also created guidance for ECHO investigators to account for cohort differences in limits of detection (LOD) and the proportion of values below LOD, availability of machine-read values, and other statistical analysis issues that arise when pooling bioassay data. For example, the DAC developed an approach to facilitate combining data when different measures are available to account for urinary dilution (i.e., specific gravity or creatinine) [49].

The heterogeneity of the contributing cohorts/sites presents an additional challenge. As the cohorts had different years of enrollment, conclusions drawn from ECHO studies may be impacted by secular trends in environmental chemical exposures, prevalence of childhood conditions such as obesity, changes in clinical recommendations in pediatric practice, and surveillance and detection of neurobehavioral and other common disorders. While this heterogeneity is not itself a limitation, it complicates the interpretation of results, particularly from studies that incorporate a subset of ECHO cohorts/sites. Related issues exist for other types of ECHO data and the DAC has employed a variety of tools and approaches to deal with considerations around standardization and harmonization of complex multi-cohort data [15, 50].

Finally, while this next cycle of ECHO will allow novel investigation of environmental exposures experienced by both conceiving partners in the preconception period, these studies will be limited (by design) to individuals and couples with demonstrated fertility because eligibility is determined by participation in ECHO with a previous pregnancy. This will limit the applicability and generalizability of these studies, particularly for environmental causes of infertility and subfertility.

### Advancing policy and practice related to perinatal and pediatric chemical exposures

While papers focusing on chemical exposures from the nationwide ECHO cohort are still nascent, it is anticipated that ongoing ECHO research will have a wide-ranging impact on public health practice and policy. For example, ECHO is providing critically important biomonitoring data on pregnant people and children, and groups that have been historically excluded from research despite their status as vulnerable populations. Furthermore, a strength of the nationwide cohort is that it includes participants from a wide range of racial and ethnic groups across all regions of the US. This provides a unique opportunity to identify populations that have the highest levels of exposures and may be uniquely susceptible to adverse outcomes. The resulting data will be critical for developing targeted interventions aimed at maximum public health benefit.

Research leveraging the multiclass chemicals panel comprised of contemporary and emerging contaminants of concern has the potential to inform other large-scale biomonitoring studies (e.g., NHANES) [38]. The focus on emerging environmental health issues also aligns with the NIEHS strategic plan [51] and can inform policy- and decision-makers as they evaluate health risks posed by environmental toxicants. For example, in 2024 the US Environmental Protection Agency (EPA) announced a proposal to add nine PFAS to its list of hazardous constituents under the Resource Conservation and Recovery Act [52]; many of these PFAS were linked to adverse birth outcomes within ECHO [23]. Additionally, while federal regulations to date typically focus on regulating a few specific chemicals from a single class (e.g., PFAS, phthalates) [53, 54], ECHO is uniquely positioned to provide information on health effects associated with cross-class chemical mixtures, which will provide critical information on how real-world exposure scenarios impact public health. The rich ECHO questionnaire data on diet, product use, and behavioral characteristics across numerous life stages (e.g., prenatal, early childhood) may also be of interest to end-user partners and organizations. For example, the US Food and Drug Administration (FDA) has a stated goal of reducing dietary exposure to contaminants to as low as possible and ECHO can provide insights into dietary sources of chemical contaminants in U.S. pregnant people and children [55]. Finally, organizations that use empirical data to advance practices and policies that protect children from environmental hazards (e.g., the Children’s Environmental Health Network) can leverage ongoing ECHO research in that area, and also access ECHO’s extant data on many contaminants of interest (e.g., heavy metals and endocrine disruptors) through DASH.

## Conclusion

In summary, ECHO offers a wealth of chemical exposures data (both existing and planned) that are now being used to develop high-impact science on maternal-child health with the potential to inform local and national policy and practice. While ECHO-wide publications on chemical exposures are still relatively few in number, numerous analyses are underway and external researchers can access ECHO chemical exposures data through NICHD’s DASH platform.

## Data Availability

Select de-identified data from the ECHO Program are available through NICHD’s Data and Specimen Hub (DASH). Information on study data not available on DASH, such as some Indigenous datasets, can be found on the ECHO study DASH webpage. Information on published ECHO papers is available at: https://echochildren.org/echo-program-publications/.
